# Ultrasound Imaging: A Cornerstone of the Entire High-Intensity Focused Ultrasound Tumor Treatment Continuum

**DOI:** 10.3390/diagnostics16193137

**Published:** 2026-09-27

**Authors:** Yufeng Zhou

**Affiliations:** State Key Laboratory of Ultrasound in Medicine and Engineering, Chongqing Medical University, Chongqing 400016, China; yufeng.zhou@cqmu.edu.cn

**Keywords:** high-intensity focused ultrasound (HIFU), sonography, lesion monitoring

## Abstract

High-intensity focused ultrasound (HIFU) has emerged as a safe and effective tumor treatment modality, harnessing thermal effects to induce tissue coagulation, cavitation effects to cause liquefaction, or a synergistic combination of both mechanisms. As a widely adopted diagnostic tool, ultrasound imaging plays an integral role throughout the entire HIFU treatment continuum, encompassing tumor diagnosis, localization, procedural navigation, real-time intraprocedural monitoring, post-treatment outcomes evaluation, and long-term clinical follow-ups. This is attributed to its real-time imaging capability, low cost, non-ionizing nature, deep tissue penetration, and satisfactory resolution and sensitivity. Conventional B-mode sonography has been extensively employed for guiding HIFU treatment, yet it is constrained by notable technical limitations. To address these issues, a range of innovative ultrasound imaging approaches have been developed. A narrative review of the literature was conducted to systematically examine the functional roles and performance characteristics of these emerging techniques and to explore their potential for clinical translation as an integrated, multi-functional single system. Evidence demonstrates significant improvement of these novel ultrasound imaging technologies across various stages of HIFU treatment, particularly in temperature imaging and tissue characterization. With continued technological advancements and systematic integration, ultrasound imaging is poised to play an increasingly crucial role in optimizing the safety and efficacy of HIFU tumor treatment.

## 1. Introduction

Cancer ranks as the second leading cause of death worldwide, posing a significant socioeconomic challenge and driving the need for more effective integrated treatment strategies [1]. In 2018 alone, it was responsible for an estimated 9.6 million deaths, representing one in every six deaths globally [2]. The global burden of cancer continues to rise, placing immense physical, emotional, and financial pressure on individuals, families, communities, and healthcare systems. Decades of sustained scientific investment have yielded progress, leading to longer survival rates even for patients with some of the most lethal forms of cancer.

High-intensity focused ultrasound (HIFU) ablation is achieved by focusing ultrasound energy to elevate tissue temperatures above 55 °C within seconds, thereby inducing localized coagulative necrosis [3,4]. To date, the thermal ablation effects of HIFU have been the most extensively explored. According to a 2024 survey conducted by the Focused Ultrasound Foundation, regulatory authorizations have approved HIFU for 38 distinct indications, with uterine fibroids, liver tumors, uterine adenomyosis, pancreatic tumor, and prostate diseases ranking as the top five therapeutic indications [5]. Additional mechanical (e.g., histotripsy, neuromodulation, blood–brain barrier opening) and thermal effects (e.g., hyperthermia) are currently under preclinical and clinical investigation. HIFU-induced effects could be adjusted flexibly and easily by modifying ultrasound parameters. Briefly, thermal effects dominate during continuous exposure, while mechanical and cavitation effects become significant with low duty-cycle, high-power pulses. Extracorporeal systems are the most versatile, enabling the targeting of both superficial and deep structures accessible through the skin. Conversely, intracorporeal systems are specifically designed; for instance, transrectal or transurethral systems target genitourinary lesions, while interstitial designs are for localized, long-term therapy following surgical implantation. HIFU ablation is typically an incisionless outpatient procedure that can be repeated over short- or long-term intervals. Furthermore, it can be integrated with other treatment modalities to augment radio- or chemotherapeutic effects, shrink inaccessible or unresectable lesions, or even replace surgery in patients with suitable lesions or those unfit for major interventions [6].

While image guidance is critical for initial targeting and patient positioning, real-time intraprocedural monitoring is essential for treatment feedback and dose delivery control, ensuring both safety and effectiveness. Currently, two primary imaging modalities are employed for guidance: magnetic resonance imaging (MRI) and ultrasound (US) [6]. MR-guided HIFU (MRgHIFU) has been adopted across various clinical fields because of its precise intraprocedural thermometry via proton resonance frequency shift (e.g., accuracy within 1 °C) and superior spatial (e.g., 1 mm) and tissue contrast resolution. This clearly delineates target tissues, necrotic zones, and healthy tissue for treatment planning and monitoring [7]. However, MRgHIFU is associated with high costs, prolonged latency between imaging and sonication, and contraindications for certain patients, such as those with pacemakers or severe obesity. Moreover, because HIFU treatment duration usually exceeds one hour [3], the limited availability of MRI scanners poses a significant logistical constraint in most hospitals. Additionally, MRI-compatible transducers are more expensive and less readily available. US-guided HIFU (USgHIFU) was introduced to overcome these limitations and has gained widespread clinical acceptance due to its accessibility, low cost, non-ionizing nature, real-time imaging capability (e.g., 30–100 Hz frame rate), ease of operation, and satisfactory spatial resolution (e.g., 1 mm) and tissue contrast. However, B-mode ultrasound imaging suffers from low contrast between treated and untreated areas and an inability to measure temperature within the ablative ranges (55–70 °C) [8]. Consequently, monitoring during USgHIFU relies heavily on detecting grayscale changes on B-mode images. Additionally, the visualization of hyperechoic spots during treatment typically requires overheating the focal zone to generate boiling or cavitation bubbles, which can result in irregularly shaped ablation lesions. Ultimately, the lack of precise monitoring in current USgHIFU systems necessitates extensive operator training, making therapeutic outcomes highly dependent on physician expertise and experience. MRgHIFU is mainly used in the United States and European countries, while USgHIFU is prevalent in Asian countries. In clinically comparative studies, USgHIFU was found to achieve a higher complete-ablation rate, fewer cases of skin burns, and shorter treatment times than MRgHIFU [9,10].

Beyond its critical role during treatment, sonography is integrated throughout the entire continuum of tumor management, from initial diagnosis, localization, and navigation to intraprocedural monitoring, therapeutic outcomes evaluation, and clinical follow-up. To address the limitations of conventional B-mode ultrasound imaging, novel ultrasound-based imaging methods are being developed, including temperature imaging and tissue characterization of necrotic tissue utilizing parameters such as backscattering coefficient, attenuation, nonlinearity, and elasticity. In this review, we summarize the capabilities and performances of these emerging techniques and discuss their potential for clinical translation as an integrated, multi-functional imaging modality for HIFU treatment.

## 2. Sonography in HIFU Procedure

Sonography serves as a full-cycle, multi-functional foundational tool throughout the entire HIFU cancer treatment procedure, addressing core needs at the stages of tumor diagnosis, treatment planning, localization and navigation, therapy monitoring, outcome evaluation, and follow-up and prediction (see Figure 1). Conventional B-mode sonography forms the base of this workflow, while emerging ultrasound technologies (e.g., coagulation monitoring, 2D thermometry, tissue characterization) are being developed to address its limitations, with the aim of integrating them into a unified, single-system solution for safer, more effective HIFU therapy.

### 2.1. Tumor Diagnosis

Ultrasound constitutes one of the most frequently performed oncologic examinations, and its popularity and applications continue to grow. Recent advancements in sonography have demonstrated unique advantages in early tumor screening, staging, and localization. Specifically, contrast-enhanced ultrasound (CEUS) and ultrasound molecular imaging utilize microbubbles (MBs), nanobubbles (NBs), and acoustic vaporization droplets (AVDs) to target vascular biomarkers, significantly enhancing tumor visualization with superior sensitivity and specificity. Multimodal ultrasound (MU), which integrates various techniques such as elastography, Doppler imaging (both duplex and color), and automated breast volume scanning (ABVS), has emerged as a powerful tool for improving tumor detection accuracy and providing critical real-time information to support clinical decision-making [11]. For instance, MU demonstrated a sensitivity of 97.85% in diagnosing benign and malignant liver tumors, significantly outperforming conventional sonography (82.56%), CEUS (92.39%), and shear wave elastography (SWE, 87.14%) [12]. Concurrently, the development of nanomedicine—leveraging polymer, metal, and metal oxide nanoparticles for their high safety, cost-effectiveness, and biocompatibility—has been synergistically integrated with ultrasound technology to diagnose various malignancies, including brain, breast, lung, liver, and pancreatic cancers [13]. Furthermore, multimodal imaging strategies that combine sonography with MRI, computed tomography (CT), and positron emission tomography (PET) have proven effective in overcoming the inherent limitations of individual modalities. For example, in diagnosing suspected ovarian tumors, sonography and MRI serve as the primary and secondary imaging modalities, respectively.

### 2.2. Treatment Planning

The primary goal of treatment planning for HIFU tumor ablation is to ensure a safe, precise, and effective non-invasive procedure (see Figure 2). This is achieved by accurately defining the target region, analyzing the acoustic path, and calculating the required thermal dose. In this context, the acoustic attenuation of multi-layered heterogeneous tissues within the focusing angle—a key factor influencing energy loss toward the focal region and, ultimately, treatment outcomes—has been quantified non-invasively and in situ based on the acoustic ray and energy loss models [14]. This was accomplished utilizing echoes from a large boiling bubble generated at the focus as a strong internal reflector. Measured acoustic attenuation coefficients of ex vivo porcine tenderloin and bovine heart at 0.97 MHz were reliable and consistent with values reported in the literature. Furthermore, significant correlations have been observed between preoperative echo amplitudes from the focus and the resulting HIFU-induced lesion areas (*R* = 0.833 for porcine tenderloin and *R* = 0.784 for bovine liver) [15]. Treatment planning that prioritizes sonication spots in descending order of echo amplitudes has demonstrated improved efficiency in ex vivo settings compared with conventional raster scanning. In another implementation, treatment planning was performed by manually segmenting a B-mode ultrasound image and computing a sonication pattern based on a user-defined region of interest (ROI) [16]. To further enhance the efficacy and safety of HIFU treatment, computer simulation has been employed. Using the Rayleigh Sommerfeld diffraction integral and angular spectrum approach, the acoustic field produced by either a concave or phased array transducer was calculated, while the perfusion-modified Pennes bioheat transfer equation was solved to predict the induced temperature field. A prospective single-institution study (KCT0003586) involving 66 female participants (median age: 44 years) with focal uterine adenomyosis compared two planning strategies: one using empirical HIFU parameters for uterine fibroids (Group A) and the other using computer simulation guided planning (Group B) [17]. Results showed that Group B exhibited a significantly higher frequency of clinically effective dysmenorrhea improvement index (DII) sustained up to 3 years post-treatment (*p* = 0.025), as well as greater adenomyosis volume shrinkage ratio (AVSR) on MRI at 3 months (*p* = 0.001), highlighting the potential benefits of simulation-based treatment planning.

### 2.3. Localization & Navigation

The primary role of localization and navigation in USgHIFU ablation is to ensure that therapeutic energy is precisely and safely delivered to the target region. This requires several key capabilities: (1) accurate spatial localization of the target based on real-time ultrasound imaging, potentially fused with preprocedural multimodal images (e.g., MRI/CT), to determine tumor boundaries and relationships to critical structures; (2) real-time beam navigation and tracking to compensate for organ motion (e.g., respiration) and dynamically adjust the HIFU focus; and (3) reliable spatial registration and coordinate mapping between the sonography and the HIFU coordinate systems. In essence, the localization and navigation system serves as the bridge between what is visualized in the image and where the energy is applied. Its precision and real-time responsiveness directly determine the accuracy, safety, and ultimate efficacy of HIFU treatment.

#### 2.3.1. MRI-Ultrasound Image Fusion

Modern, precise clinical decision-making relies on high-quality visual evidence. While real-time sonography offers the advantage of live guidance, it is inherently limited by lower spatial resolution and soft tissue contrast. Conversely, MRI, widely utilized for detailed tumor diagnosis, provides excellent anatomical delineation but lacks real-time capability. The fusion of these two modalities combines their complementary strengths, enabling a diagnostic assessment that surpasses what either can achieve independently (see Figure 3). Recent advancements in machine learning (ML) have demonstrated that convolutional neural networks (CNNs), encoder–decoder architectures, and transformer-based systems can effectively collaborate to synthesize high-quality, multi-channel fused images, thereby enhancing cancer identification [18]. By integrating comprehensive networks (e.g., CNN, U-Net, GAN) with Vision Transformer modules through systematic attention protocols and synthetic data creation pathways, secure and reliable real-time medical navigation systems can be developed [19]. This deep-learning-driven fusion approach is establishing itself as a cornerstone of next-generation diagnostic imaging. In clinical practice, the fusion of preoperative MRI with intraoperative sonography has been successfully implemented to guide HIFU treatment of uterine fibroids, utilizing 3D nnU-net models for image registration and segmentation [20,21]. This methodology has achieved Dice similarity coefficients (DSC) of more than 90% for segmenting key anatomical structures, such as the uterus and fibroids, and has facilitated efficient and precise target tracking in more than 90% of cases. Consequently, this smart navigation system, founded on MRI-US image fusion, offers an efficient and automated solution for HIFU treatment, substantially improving procedural accuracy and operational efficiency.

#### 2.3.2. Interference Removal

In USgHIFU, the simultaneous use of ultrasound for both therapeutic energy delivery and real-time imaging guidance is typically hindered by substantial mutual interference. This interference occurs because HIFU pulses generate intense fundamental and harmonic signals whose frequency spectrum overlaps with that of diagnostic sonography, leading to radio-frequency cross-talk and over-saturation of the B-mode ultrasound image. To enable reliable real-time guidance, this interference must be effectively suppressed. Several advanced strategies have been developed to address this challenge. Singular value decomposition (SVD)–based filtering can restore ultrasound harmonic images from HIFU interference without introducing undesirable spectral distortion [22]. Pulse inversion (PI) imaging has been shown to effectively suppress HIFU-induced artifacts, enabling visualization of echogenicity changes in the bovine liver even under respiration-like motion conditions (see Figure 4) [23]. An interleaved method for harmonic motion imaging guided focused ultrasound (HMIgFUS) demonstrated strong robustness against interference during HIFU ablation monitoring, achieving higher contrast-to-noise ratios (CNRs) compared with notch filtering and FUS-net approaches [24]. A frequency-domain robust principal component analysis (FRPCA) method successfully separated HIFU interference from contaminated B-mode images in both ex vivo and in vivo experiments [25]. Compared to conventional notch filtering, FRPCA improved the structural similarity (SSIM) index by 8.9%, thereby enabling improved dynamic visualization. A deep learning (DL) approach, specifically a diffusion implicit (HIFU-Diff) model, suppressed low-resolution HIFU interference and reconstructed high-resolution images through a series of forward diffusion and reverse sampling steps [26]. This network outperformed notch filtering and 1D FUS-Net in reconstructing interference-free images under various ex vivo and in vivo conditions. A single-transmit bipolar sequence with minimum-peak-sidelobe (MPS) coding, whose duration is an integer multiple of the HIFU waveform period, not only removed acoustic interference in the presence of tissue motion but also provided better image quality in sonography than pulse inversion subtraction (PIS) and Golay decoding methods [27]. Furthermore, the interference-free images reconstructed by these methods can also support quantitative acoustic applications, such as attenuation imaging, confirming that the underlying acoustic properties of the tissue are preserved in the processed images.

### 2.4. Monitoring

The purpose of real-time, quantitative monitoring in HIFU cancer treatment is to provide a quantitative assessment of tissue changes, thermal dose, and bubble activity during ablation to ensure treatment efficacy and safety. This requires the real-time analysis of backscattered pulse-echo signals and/or the reconstruction of new imaging using ultrasound radio frequency (RF) data for each individual ablation site, thereby enabling precise dose control and feedback during the procedure.

#### 2.4.1. Lesion Detection

USgHIFU faces challenges in achieving precise, quantitative monitoring, which often leads to unpredictable ablation shapes. Three primary sonography-based strategies are currently employed for lesion detection: (1) advanced analysis of ultrasound RF data, (2) assessment of tissue mechanical responses, and (3) evaluation of changes in scatterer concentration and distribution within the target.

The first strategy relies on advanced RF data analysis. High frame rate (77–130 Hz), high-frequency (55 MHz) B-mode ultrasound imaging has been used to monitor tissue necrosis and gas-body activity in ex vivo porcine cardiac tissue specimens during HIFU ablation [28]. Temporal maximum integrated backscatter (IBS) with a 12 dB threshold provided the best lesion identification, yielding a receiver operating characteristic (ROC) curve area of 0.96. Frame-to-frame echo decorrelation successfully identified and tracked transient bubble activities. Macroscopic cavities on the order of millimeters formed when the initial bubble expansion speed exceeded 0.8 mm/s. An integrated and dynamic monitoring technique that uses specific sequences of HIFU exposures and RF data acquisition, followed by post-treatment algorithms, can separate cavitation from thermal lesions and display them simultaneously [29]. A Swin-Unet architecture-based segmentation model distinguished hyperechogenicity in B-mode ultrasound imaging as ablated regions, achieving 93% accuracy on chicken breast [30]. A feed-forward neural network, whose features included changes in tissue’s attenuation coefficient, integrated backscatter, Nakagami distribution scaling parameter, frequency-dependent scatterer amplitudes, and tissue vibration, effectively detected HIFU-induced thermal lesions in ex vivo tissues with a minimal size estimation error (estimated 9.6 mm × 8.5 mm vs. actual 10.1 mm × 9 mm) [31]. According to the varying numbers of intrinsic mode functions (IMFs) produced by the empirical mode decomposition (EMD), ultrasound signals can be adaptively compressed in the source domain and reconstructed in the receiver domain. This compressive sensing based on empirical mode decomposition (CS-EMD) provides a feasible approach for monitoring HIFU-induced thermal lesion dimensions using various wavelet transforms and is extendable to higher-dimensional data like 3D and 4D ultrasound [32]. The twinkling artifact (TA) induced by the acoustic radiation force and consequent tissue vibration during Doppler imaging has been employed for real-time monitoring of HIFU-induced lesions in ex vivo porcine tissue samples, yielding less than 5% error in depth and width measurements for depicting coagulated tissue dimensions (see Figure 5) [33]. High-frequency M-mode ultrasound imaging has been used to measure spatiotemporal changes during HIFU treatment to identify coagulation necrosis [34], achieving a reduced gray-scale intensity of 7.8 ± 3.3 dB, an accuracy of 85 ± 8%, an ROC curve area of 0.91 ± 0.04, and a lesion depth error of 2.3 ± 2.4 mm [35].

The second strategy leverages tissue mechanical properties. SWE was utilized to monitor the thermal responses of targeted tissue during HIFU treatment, and PI imaging was integrated to suppress HIFU-induced interference, thereby improving its accuracy [36]. Progressive stiffening due to tissue coagulation (from 2.53 ± 0.32 kPa to 16.15 ± 1.84 kPa), improved uniformity (±13.55 kPa without PI vs. ±3.65 kPa with PI), and reduced variability in the rate of change (±5.00 kPa/min without PI vs. ±3.56 kPa/min with PI) were demonstrated in ex vivo experiments. Thus, PI-SWE provided robust, interference-free monitoring with high sensitivity to tissue mechanical changes. Axial strain elastography (ASEs) and axial-shear strain elastograms (ASSEs) provided accurate location information and lesion size at low power output [37]. However, at high power, cavitation bubbles influence lesion appearance more in ASEs than in ASSEs. Transient ASSEs reconstructed using high-speed sonography in a gel phantom and ex vivo porcine kidney and liver samples confirmed the presence of substantial axial shear strain in the gap zone between adjacent lesions, which may contribute to cancer relapse [37,38]. A steady decrease in displacement, compressive strain, and relative change in focal phase shift (φ) was observed in ex vivo canine liver specimens using harmonic motion imaging for focused ultrasound (HMIFU), attributable to an overall increase in tissue stiffness [39]. During boiling, a reverse lesion-to-background displacement contrast was detected, indicating potential changes in tissue absorption, geometry, and/or mechanical gelatinization or pulverization. A fast beamforming algorithm for image reconstruction using a graphics processing unit (GPU)-based delay-and-sum (DAS) method and a sparse-matrix operation with real-time feedback was implemented for HMIFU, achieving frame rates up to 15 Hz—a 100-fold increase over conventional central processing unit (CPU)-based processing—and showing a consistent average focal displacement decrease of 46.7 ± 14.6% during thermal coagulation [40].

The third approach exploits alterations in scatter concentration and distribution. Pathological sections from HIFU-ablated porcine liver tissues reveal significant differences in scatterer concentration and distribution between normal and denatured tissues. Entropy parameters of ultrasound backscattered signals were extracted to reconstruct entropy imaging for HIFU thermal lesion detection [41]. Contrast-to-speckle ratio (CSR) values in entropy images were significantly higher than those of conventional sonography at acoustic powers ranging from 30 to 110 W in ex vivo porcine muscle samples (e.g., a 3.4-fold increase at 110 W, *p* < 0.05). Compared with in situ B-mode sonography, weighted ultrasound entropy (WUE) imaging exhibited higher image quality in indicating lesion formation under various acoustic power levels (174–308 W), with a 39.2–53.4% improvement in the CNR in both ex vivo and in vivo experiments and a strong correlation (*R* = 0.84) between the damage area estimated from WUE images and that measured from dissected ex vivo tissue samples [42]. Ultrasonic Nakagami imaging, which traces changes in the Nakagami parameter, was employed to identify thermal lesions in ex vivo porcine liver tissues that are difficult to visualize using conventional B-mode sonography due to the absence of apparent bubble formation. This method offers a more accurate estimation of lesion size and position while maintaining low computational complexity [43]. The sub-resolvable effect aids thermal lesion identification, and precision is related to the Nakagami values chosen for boundary detection [44]. Thermal lesion sizes assessed using ultrasonic Nakagami images correlated well with gross-pathology images (*R* ≥ 0.9 ex vivo and *R* ≥ 0.85 in vivo). Moreover, the mean CNR values of the ultrasonic Nakagami images were 1.29 dB (ex vivo) and 0.80 dB (in vivo), significantly higher than those for B-mode images (*p* < 0.05) [45]. Ultrasound Nakagami-m parametric imaging can evaluate and differentiate between thermal coagulation and cavitation erosion induced by HIFU in bovine serum albumin (BSA) phantoms and ex vivo porcine livers, highlighting the detrimental shadowing effects of bubbles [46]. The thermal and mechanical lesion sizes estimated using this technique were all closer to the actual lesion sizes than those depicted in conventional sonography. To comprehensively characterize HIFU-induced tissue damage, ultrasonic Nakagami imaging, horizontally normalized Shannon entropy (hNSE) imaging, and differential attenuation coefficient intercept (DACI) imaging were simultaneously reconstructed from ultrasound backscattered envelope data [47]. Nakagami imaging excels at distinguishing coagulative necrosis from cavitation erosion, while hNSE and DACI imaging provide complementary assessment of scatterer microstructural changes and tissue attenuation properties, respectively. The combined use of these modalities can effectively mitigate the imaging artifacts caused by bubble shadowing, enabling more accurate delineation of both thermal and mechanical lesion boundaries than conventional B-mode imaging alone.

To further compensate for the small sampling volume inherent in these quantitative imaging techniques, a complementary strategy based on the analysis of pulse-echo signals from the focused ultrasound (FUS) transducer itself has been implemented. By driving the transducer in a dual-mode capacity, variations in the received echo amplitudes were captured and analyzed in both the time and frequency domains. Coagulation prediction utilizing the FUS transducer’s echo amplitudes achieved superior accuracy (94.4% vs. 50%), sensitivity (92.9% vs. 35.7%), and negative prediction (80% vs. 30.8%), but similar specificity (80% vs. 100%) and positive prediction (100% vs. 100%) compared with conventional sonography in in vivo goat experiments involving ablation of the liver, kidney, and leg muscles [48].

#### 2.4.2. Thermometry

A critical challenge for USgHIFU is the lack of quantitative, real-time temperature mapping in the focal region, akin to MRI thermometry [8]. To bridge this gap, several ultrasound-based thermometry techniques have been developed. An algorithm for dense speed-of-sound shift imaging (DSI) simultaneously incorporated information from the entire field of view by combining dense optical flow with inverse problem regularization, which accelerated computation and inherently enforced spatial agreement between pixels [49]. In ex vivo chicken breast, this method measured temperature changes between 3 °C and 15 °C with an error of less than 2 °C. Thermal strain during HIFU sonication has also been measured ultrasonically and converted to temperature using effective tissue-dependent coefficients [50]. However, its accuracy degraded over longer exposure and wider temperature ranges. The second harmonic of the change in backscattered energy (CBE_h2_) and the combined fundamental-second harmonic CBE (CBE_comb_) have been used to produce 2D temperature maps, detect localized heated regions, and control temperature rise up to 43 °C [51]. Nonetheless, the aforementioned methods are not feasible for the temperature range required in HIFU therapy. By neglecting the effects of multiple scattering, the ultrasound backscattered energy (ΔBSE) from a set of identical particles is proportional to the number of scatterers per unit volume, the back-scattering cross-section, and the structure factor that accounts for structural correlations in dense media. Variations in ΔBSE were used to monitor temperature increases in ex vivo bovine liver up to 90 °C during HIFU treatment, showing a linear relationship (*R* = 0.94, *p* < 0.001) at the frame rate of conventional B-mode sonography with an acceptable accuracy of 5 °C [52]. Irreversible changes in ΔBSE after cooling were observed only when the maximal temperature exceeded 70 °C.

An alternative strategy for ultrasonic thermometry integrates ultrasound real-time RF data with MRI-measured temperature fields using artificial intelligence (AI) technologies. An end-to-end deep neural network, termed the Breath-guided Multimodal Teacher-Student (BMTS) model, reconstructed 2D temperature distributions in deep organs with an average error of 0.8 °C, a processing speed of 0.37 s, and a maximum measurable temperature of 67 °C [53]. The multimodal attention multiscale teacher-student knowledge distillation model (MMAM-TSD) elucidated the interaction between HIFU and complex heterogeneous biological media, enabling real-time, precise temperature measurements in the HIFU focal region (see Figure 6) [54]. Quantitative evaluation demonstrates its robust performance across ex vivo and in vivo (leg and kidney) experiments, with a mean temperature-estimation accuracy of 0.56 ± 0.14 °C, a spatial resolution of 0.195 mm/pixel, and superior uniformity (coefficient of variation 21% lower than that of MRI).

#### 2.4.3. Cavitation Detection

Cavitation plays a pivotal role in HIFU therapy, particularly for soft tissue disintegration via histotripsy. Currently, acoustic cavitation is under investigation for minimally invasive therapeutic techniques across a broad spectrum of pathologies. Accurate localization and real-time monitoring of bubble activity are essential for the closed-loop control of HIFU-induced cavitation effects, thereby ensuring both safety and efficacy. Given the stochastic nature of cavitation, a real-time spatial monitoring system is indispensable. Residual cavitation bubbles function as nucleation sites, facilitating subsequent cavitation events, and act as strong scatterers, resulting in acoustic shadowing.

Bubble dynamics have been monitored via acoustic emissions captured by a 15-MHz passive cavitation detector (PCD) aligned confocally and coaxially with the HIFU transducer. Studies in ex vivo bovine liver demonstrated that the successful formation of HIFU lesions correlates strongly with the presence of high-order harmonics in the spectrum, achieving an accuracy of 80%, a sensitivity of 75.6%, and a specificity of 74.2% [55]. While PCD offers a low-cost, real-time approach for monitoring clinical HIFU treatments, it is limited in proving the spatial distribution of activity within the focal region. In the context of sonography guidance, support vector machine (SVM) algorithms have been utilized to rapidly and accurately detect cavitation occurrence. Subsequently, an improved watershed algorithm was employed to extract cavitation regions, with images reconstructed using the total focusing method based on sparse array elements [56]. Furthermore, an ultrafast active cavitation imaging method, which combined plane wave transmission, minimum variance beamforming, and coherence factor weighting, has demonstrated a relatively high signal-to-noise ratio (SNR) and good spatial-temporal resolution for monitoring residual cavitation bubbles in histotripsy [57]. A direct correlation (*R* = 1.0365) was identified between histotripsy lesion area and the inner residual bubble area. Conventional harmonic imaging, such as PI, often suffers from insufficient contrast due to the non-uniform sizes of cavitation bubbles. However, superimposing a lower-frequency pulse (sHIFU) from the HIFU transducer with a higher-frequency pulse (imaging pulse) from the imaging probe resulted in approximately 23 dB higher contrast compared to PI [58]. Peak contrast was observed at sHIFU pressure of 4.6 MPa, where a high-pass filter (passband frequency of 2.5 MHz) maximized detection by suppressing sHIFU reflections while preserving the imaging bandwidth. Dynamic mode decomposition (DMD) has been applied to in vivo Doppler power map of cavitation bubbles in porcine liver using k-means clustering and an image contrast-based algorithm [59]. The DMD-enhanced power Doppler map effectively visualized bubble distribution with high contrast, and the Doppler power levels correlated with the severity of cavitation-induced tissue damage. Moreover, bubble decay rates derived from DMD correlate with tissue damage, with slower rates indicating more severe injury (see Figure 7). Passive acoustic mapping (PAM), adapted from seismic imaging, has been employed for cavitation localization using time-exposure acoustic or DAS method [60]. PAM weighted with a phase coherence factor (PAM-PCF) beamforming enables the localization of the focal cavitation point in two perpendicular imaging planes, validated in both water and agar gel [61]. To enhance the axial resolution of PAM, cross-spectral matrix fitting (CMF) was adapted, reducing the axial full width at half maximum (FWHM) by factors of 3 and 5 compared to the robust capon beamformer (RCB) and DAS, respectively, while improving CNR by more than 15 dB over RCB [62]. Finally, for resolving the inverse problem for cloud-shaped cavitation sources, the sparse Total Variation (spTV) method was found to be superior to Elastic Net (ElNet).

### 2.5. Therapeutic Outcomes Evaluation

Accurate evaluation of HIFU ablation outcomes is critical to ensure complete tumor eradication and minimize local recurrence. CEUS performed intraprocedurally and immediately after treatment plays a pivotal role in assessing the extent and boundaries of thermal lesions by dynamically visualizing microvascular perfusion in a cost-effective manner. Complete absence of contrast enhancement within the targeted zone indicates successful coagulative necrosis, whereas residual enhancement suggests residual viable tumor tissue requiring retreatment. Thus, immediate retreatment can be performed to minimize cancer recurrence [63]. Intraprocedure CEUS correlated well with post-HIFU MRI without severe complications observed in clinical applications [64]. It can identify residual disease after focal HIFU, showing 72.7% agreement with early multiparametric MRI (mpMRI) and an area under the curve (AUC) of 0.881 [65]. To complement the hemodynamic information, passive elastography has emerged as a valuable tool for characterizing the mechanical properties of ablated tissue. Unlike conventional shear-wave elastography, passive elastography utilizes the intrinsic tissue vibrations or acoustic radiation force induced by the HIFU process itself to map tissue stiffness. Because thermal ablation induces protein denaturation and subsequent tissue hardening, passive elastography provides a quantitative stiffness map that correlates with the extent of the thermal lesion, achieving a CNR of 9.2 dB when delineating HIFU-induced lesions of various sizes in in vivo porcine livers [66].

### 2.6. Follow-Up and Prediction

Although MRI remains the reference standard for evaluating HIFU outcomes, CEUS serves as a valuable complementary modality in follow-up assessment, primarily owing to its low cost and ease of operation. Contrast-enhanced transrectal ultrasound (TRUS) has been employed for follow-up of prostate cancer patients after HIFU hemiablation at 3, 6, and 12 months [67]. Contrast-enhanced TRUS clearly delineated the ablation defect as a sharply marginated nonenhancing zone in all patients, identified suspicious foci of recurrent enhancement, and demonstrated good correlation with mpMRI and biopsy histologic findings. Image fusion of CEUS and mpMRI (e.g., T1-, T2-, and diffusion-weighted MRI) is a cost-effective and feasible technique for assessing perfusion within the ablation zone.

Nomograms are predictive tools that integrate multiple clinical and imaging features to provide personalized risk stratification and facilitate clinical decision-making. They have proven effective across various medical fields, including oncology and radiology. A nomogram based on sonographic features was developed to predict the efficacy of HIFU ablation in patients with uterine fibroids, achieving an AUC of 0.900 (95% CI 0.840–0.901), a sensitivity of 0.759, and a specificity of 0.818 [68]. The calibration curve demonstrated good agreement between observed and predicted outcomes, and decision curve analysis (DCA) confirmed its clinical utility and broad applicability. When combined with the oxytocin-induced arterial-phase perfusion delay time (APDT) on CEUS, the nomogram achieved an AUC of 0.884 (95% CI: 0.803–0.965), a sensitivity of 92.7%, and a specificity of 76.2% [69]. Collectively, these findings suggest that robust prediction of HIFU ablation outcomes is feasible.

Overall, the pros and cons of current sonography advancements across the entire HIFU treatment workflow are summarized in Table 1.

## 3. Discussion

Sonography plays a pivotal role in HIFU cancer treatment. Currently, clinical USgHIFU predominantly relies on conventional sonography for intraprocedural feedback. Consequently, the development of new monitoring methods that utilize ultrasound hyperechogenicity to enable more accurate and efficient treatment administration is highly desirable. While recent advances over the past two decades have addressed some limitations encountered in clinical practice, it is important to acknowledge that these methods remain in the early stages of investigation. Indeed, several are still confined to laboratory settings involving phantom, ex vivo tissue, and animal studies, and have not yet received clinical approval.

The clinical adoption of USgHIFU poses unique technical challenges. For instance, in hepatic tumor ablation, the interposed rib cage and continuous respiratory motion are principal obstacles. Contemporary ultrasound imaging navigates rib acoustic shadowing through several strategies: real-time sonography and 3D/4D scanning identify intercostal acoustic windows to guide patient positioning [70]; automatic rib segmentation enables rib-sparing aperture control by shutting off phased array elements whose beams would otherwise strike bone; and phase-aberration corrections restore a sharp focus despite residual rib-edge effects [71,72]. To address respiratory motion, modern systems employ ultrafast or high-frame-rate imaging to reduce motion blur and use speckle tracking to estimate target displacement in quasi-real time. Compensation is achieved by gating sonication to stable end-exhalation windows or by electronically steering the HIFU focus to follow the moving tumor using predictive filters that anticipate the target’s future position. Interleaved imaging and therapy updates further refine target location between pulses, while 4D motion models or US/MRI fusion provide additional guidance when the tumor itself is partially shadowed by ribs, thereby maintaining accurate ablation despite rib obstruction and breathing-induced liver motion [73].

The current era has witnessed the rapid development and widespread application of AI technologies, from which the HIFU field has greatly benefited. Increased research efforts and clinical translation are expected to revolutionize the entire treatment workflow. Key expectations include reducing reliance on operator experience, enabling accurate and predictive treatment planning, enabling sensitive treatment monitoring, and automatic parameter settings to achieve consistent outcomes across diverse patient populations. Ultimately, the goal remains consistent: improving the efficacy, safety, and effectiveness to enhance patient outcomes. While computer-simulated optimized treatment planning for uterine adenomyosis has yielded promising short- and long-term results [17], adverse events such as buttock skin burns and sciatic nerve pain requiring treatment were observed in two participants. These findings suggest that the complex bioeffects induced by HIFU pulses and their interaction with biological tissues require further investigation. Current simulation models need to incorporate more comprehensive physical and biological phenomena. Moreover, real-time, accurate, and quantitative monitoring provides a solid foundation for subsequent closed-loop feedback control of sonication, thereby mitigating the risk of adverse events such as skin burns, post-procedural pain, nerve injury, local edema, and unintended thermal damage to adjacent organs or structures. Most complications are transient and manageable with appropriate patient selection and technique; major adverse events, including bowel injury, acute renal failure, deep vein thrombosis, and pubic symphysis injury, occur at a rate of only 0.38% [74]. The primary mechanism underlying HIFU-induced adverse events is unintended energy deposition in intervening tissue due to acoustic field redistribution by cavitation bubbles and energy absorption by certain tissue (i.e., scar tissue). Selecting an appropriate acoustic window, optimizing treatment planning, and carefully monitoring sonographic changes during treatment contribute to a satisfactory low complication rate, which has continuously decreased since the introduction of HIFU procedures. Furthermore, AI integration is expected to extend beyond individual tasks, functioning as systematic assistance throughout the entire workflow. For instance, the optimized multi-task contrastive learning framework (OMCLF) leveraged self-supervised learning (SSL) and genetic algorithms (GA) to enhance HIFU lesion detection and segmentation by optimizing feature representations, hyperparameters, and data augmentation strategies [71]. This framework outperformed single-task methods in both classification and segmentation tasks while significantly reducing dependence on labeled data, achieving an accuracy of 93.3% in lesion detection and a Dice score of 92.5% in segmentation, surpassing state-of-the-art methods like SimCLR and MoCo. In the near future, more AI models are expected to be seamlessly integrated across all operational tasks. However, implementing AI in HIFU tumor ablation faces several practical challenges. The scarcity of large, well-annotated clinical datasets and inherent procedural variability can hinder robust model training and validation. Real-time processing demands are also critical, as AI must rapidly interpret imaging and acoustic signals for temperature monitoring, motion compensation, and intraoperative feedback without introducing latency. Integration with existing HIFU platforms and sonography is technically complex and often vendor-specific, limiting interoperability and widespread adoption. Additionally, regulatory approval, clinician trust, and model interpretability remain significant barriers, as errors in ablation planning or real-time guidance could directly compromise patient safety. Generalization across different tumor types, anatomical sites, and device settings further complicates reliable AI deployment.

Beyond sonography and MRI, other imaging modalities may be incorporated into the HIFU treatment procedure. For example, multi-wavelength photoacoustic (MWPA) imaging enables monitoring of HIFU lesion formation by repeating PA imaging at multiple optical wavelengths to thoroughly sample the optical absorption spectrum [75]. With the aid of CNNs, HIFU lesions in MWPA images can be segmented with high accuracy. Although normal tissue provides little acoustic contrast in the absence of cavitation or boiling bubbles, its optical properties alter significantly during lesion formation. A photorefractive crystal-based acousto-optic (AO) sensing system, which utilizes the HIFU transducer to both generate tissue necrosis and drive the AO interaction, has been used to monitor real-time optical changes associated with thermal lesions induced in ex vivo chicken breast [76]. AO sensing can identify lesion onset and growth in real time; when used as feedback to guide exposures, it results in more predictable lesion formation. However, given the space constraints of extracorporeal HIFU systems, the clinical translation potential of these approaches requires extensive evaluation.

## 4. Conclusions

Ultrasound imaging is an indispensable, multi-functional tool throughout the entire HIFU tumor treatment workflow, encompassing initial diagnosis, treatment planning, intra-procedural navigation and monitoring, and post-treatment follow-up. Although conventional B-mode ultrasound imaging has limitations such as low contrast between treated and untreated tissues and the inability to measure temperature, emerging techniques—including contrast-enhanced ultrasound, multimodal ultrasound, MRI-US image fusion, AI-driven interference removal, and advanced RF data analysis for lesion detection—are actively addressing these gaps. These innovations improve the accuracy, safety, and efficiency of USgHIFU while reducing reliance on operator expertise. Although some approaches remain limited to phantom, ex vivo tissue, and in vivo animal studies, their feasibility has been demonstrated. With continued technical advances and systematic integration, ultrasound imaging is poised to evolve into a more robust, unified single-system solution for HIFU tumor therapy, thereby expanding its clinical utility.

## Figures and Tables

**Figure 1:**
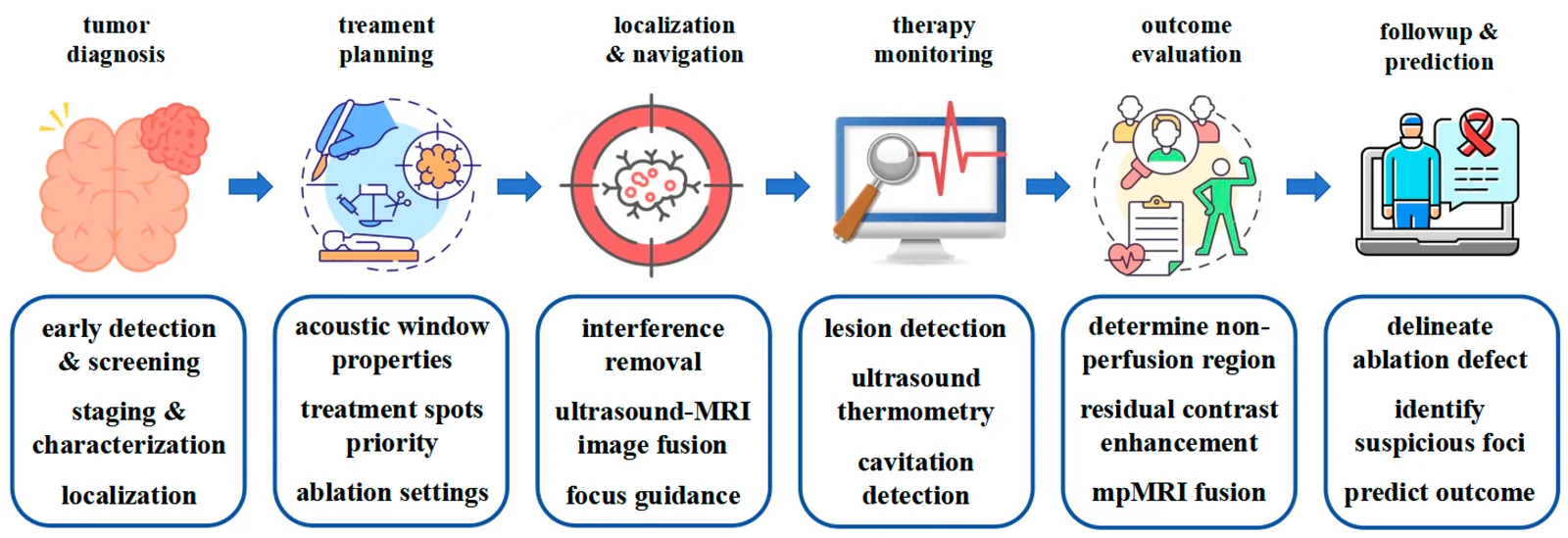
Schematic diagram of the entire HIFU cancer treatment procedure and involvement of sonography in individual states.

**Figure 2:**
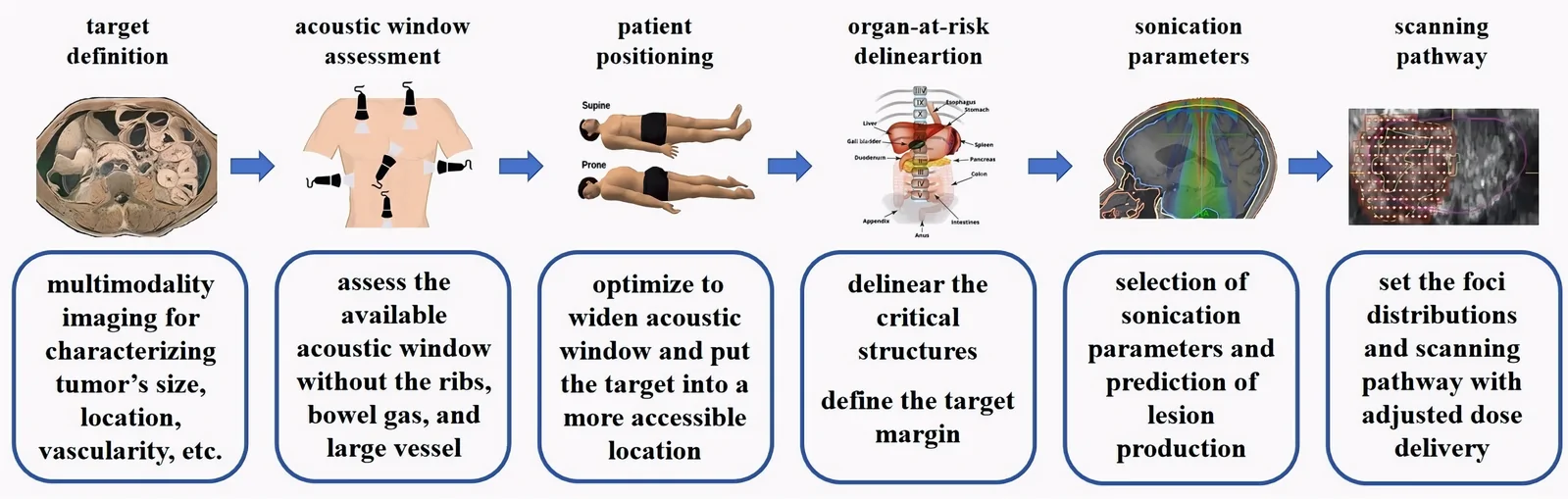
Schematic diagram of HIFU treatment planning.

**Figure 3:**
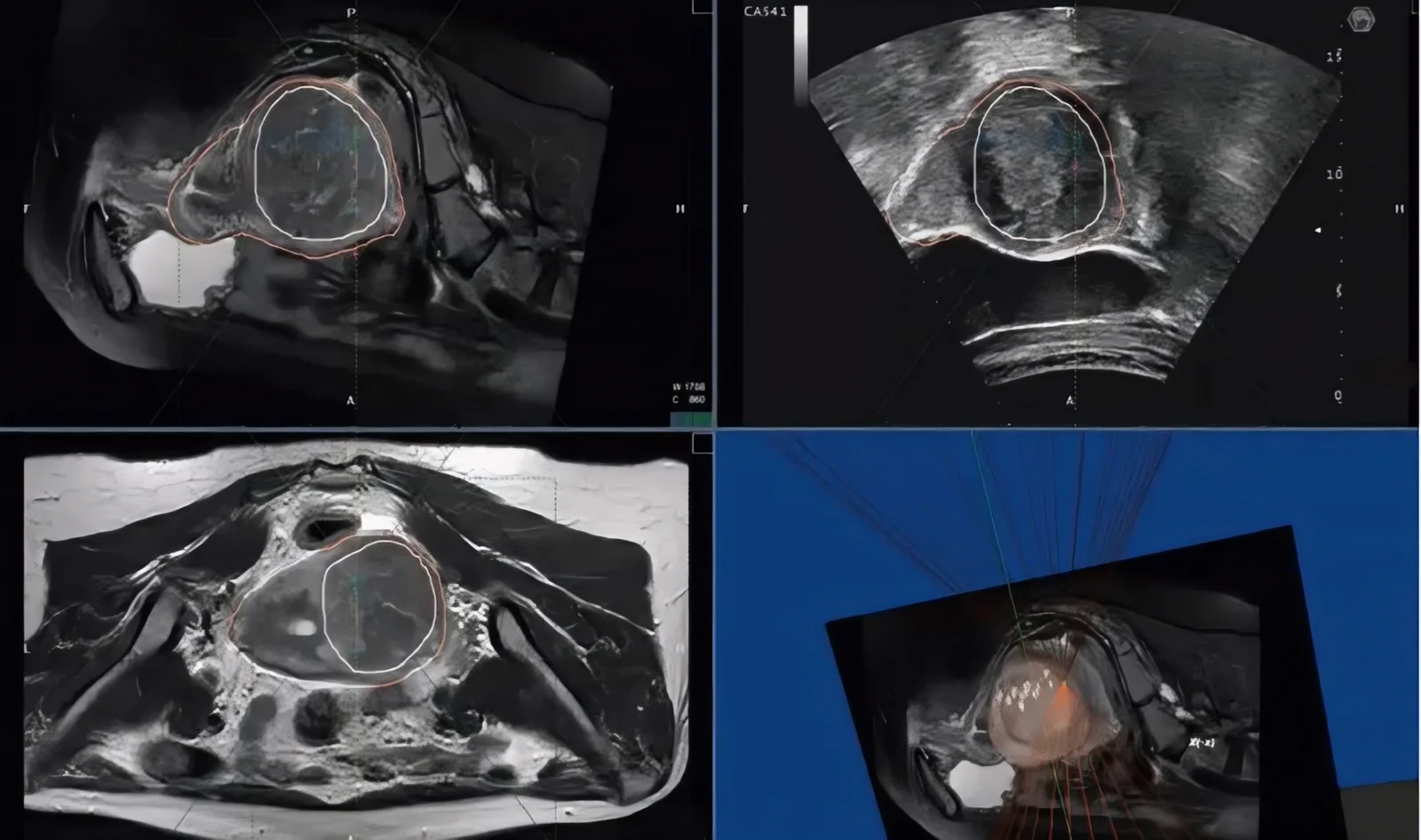
A representative case of MRI-ultrasound image fusion in the HIFU treatment of uterine fibroids.

**Figure 4:**
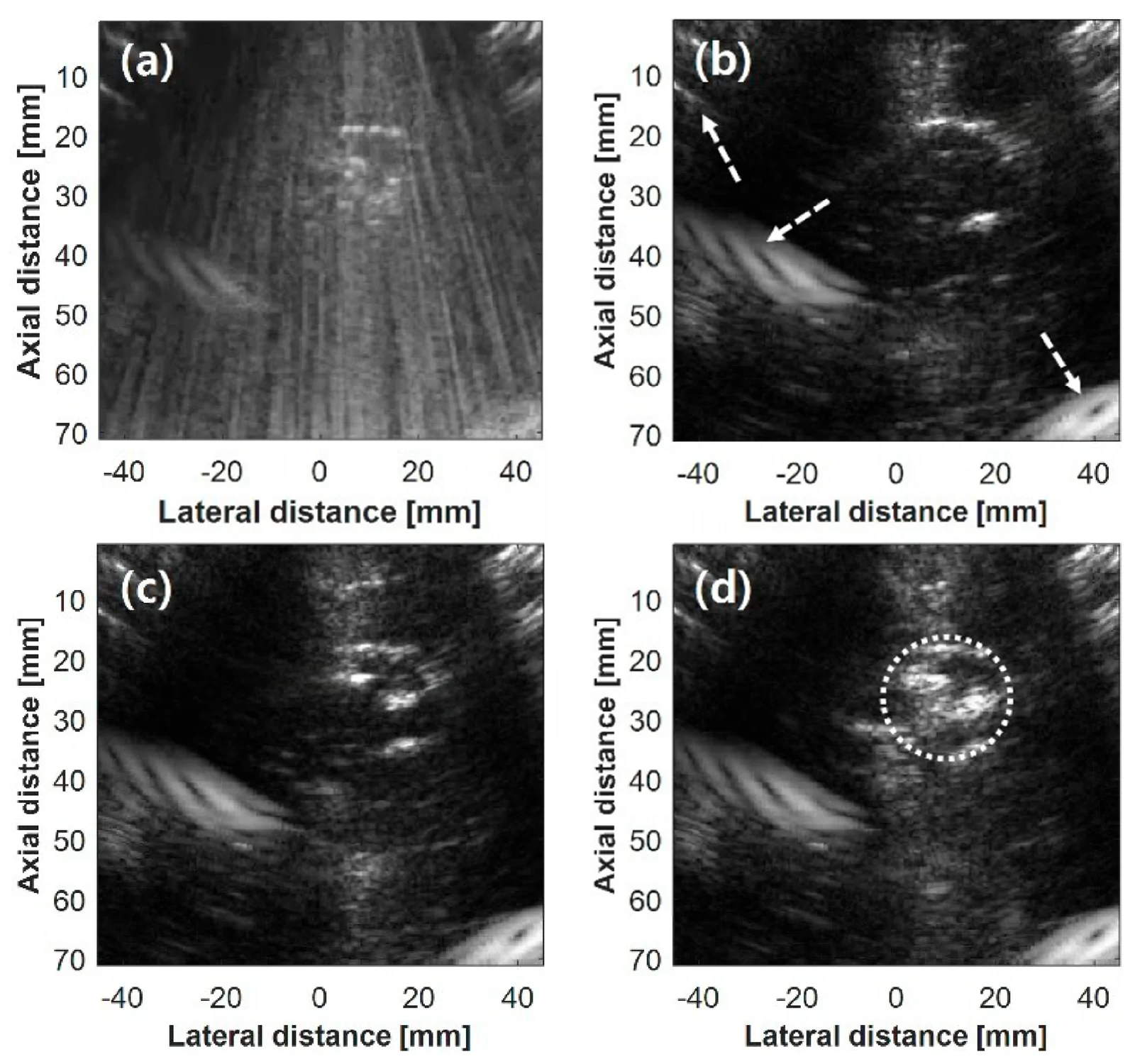
(**a**) Presence of interference in sonography during HIFU treatment of ex vivo bovine liver, and its disappearance when applying the pulse inversion method at (**b**) 0 s, (**c**) 5 s, and (**d**) 15 s after HIFU insonation. Dashed white arrows: reverberation images; dotted white circle: the predicted treatment area. Courtesy of [23].

**Figure 5:**
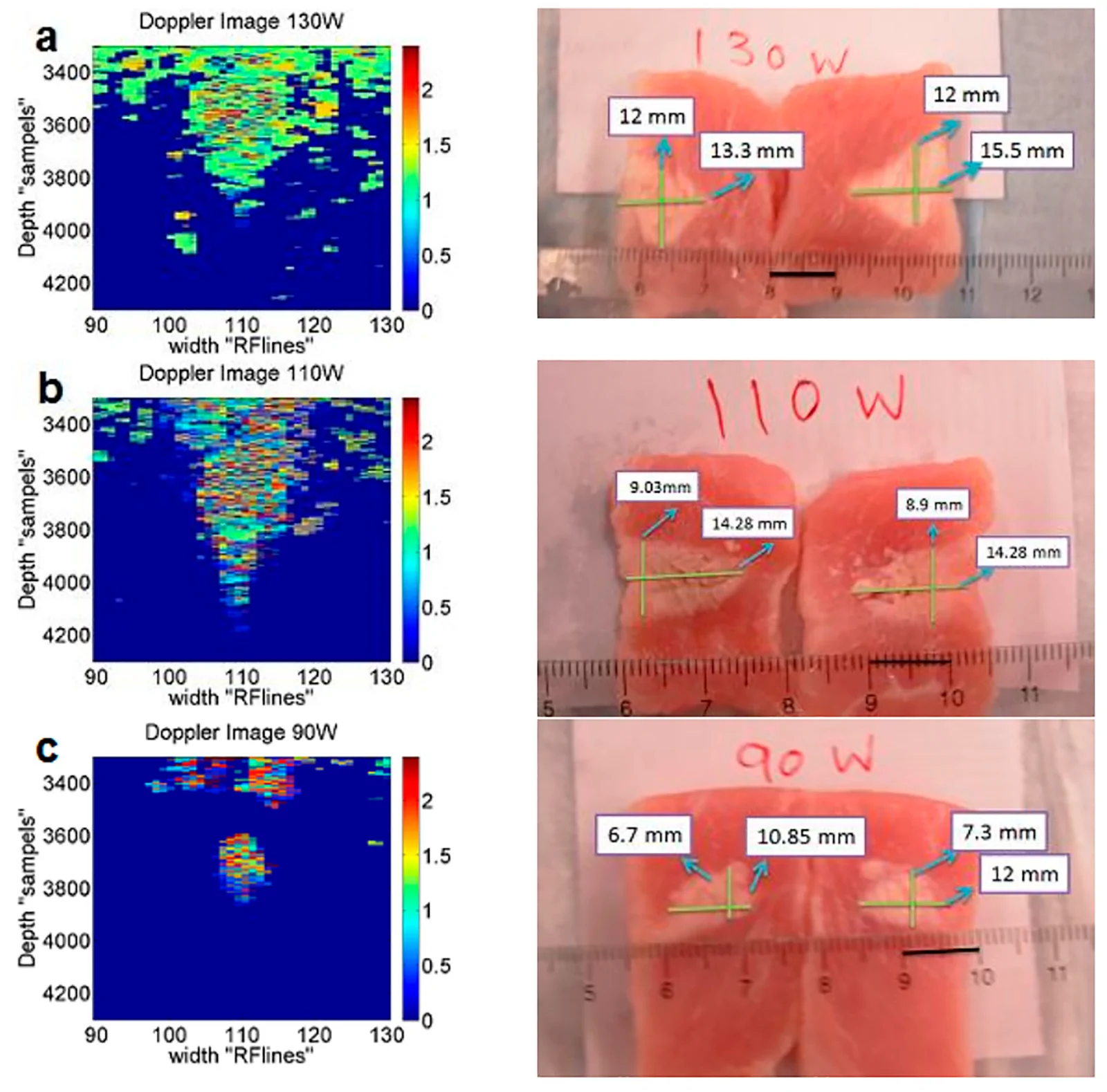
Comparison of constructed Doppler images after HIFU treatment at (**a**) 130 W, (**b**) 110 W, and (**c**) 90 W, alongside photograph of lesions in dissected ex vivo porcine muscle. Courtesy of [33].

**Figure 6:**
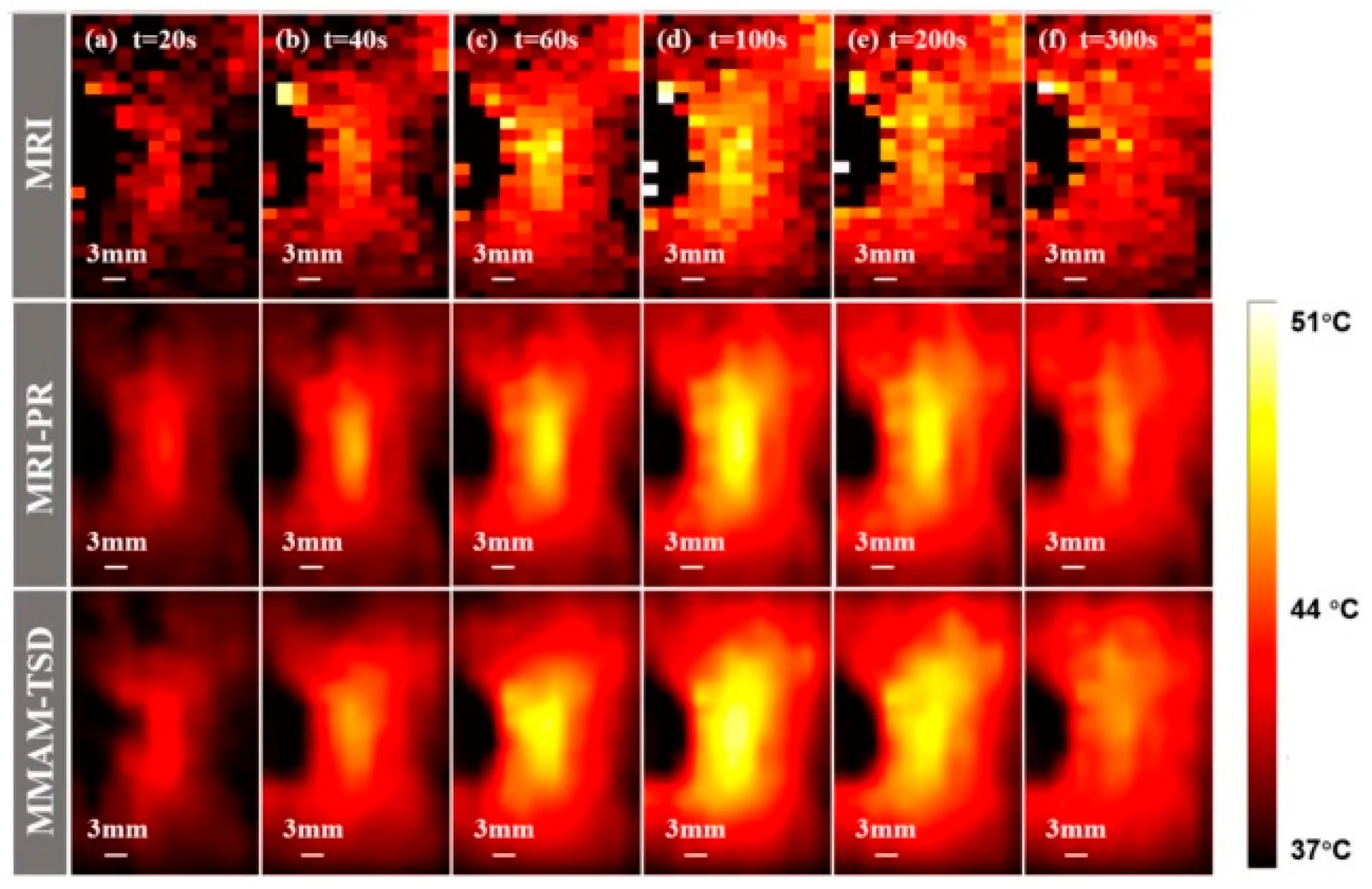
Comparison of temperature field reconstruction in an in vivo kidney utilizing MRI, MRI proton resonance frequency shift (PRF), and an end-to-end deep neural network architecture of MMAM-TSD under HIFU power of 60 W. Courtesy of [54].

**Figure 7:**
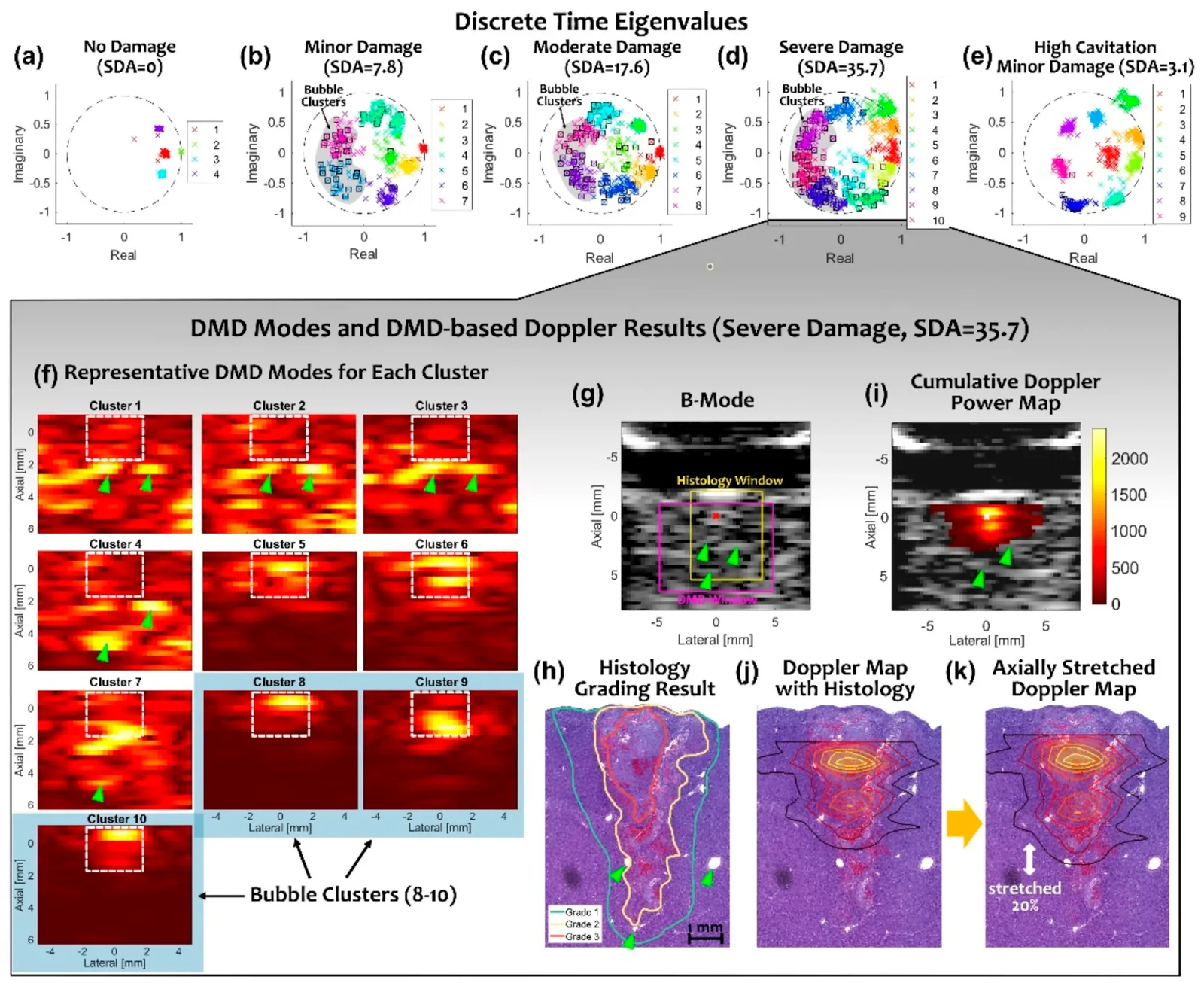
Dynamic mode decomposition (DMD) eigenvalues after HIFU treatment in porcine liver in vivo under different conditions: (**a**) no discernible damage at 170 W, (**b**) low and (**c**) moderate damage at 260 W, (**d**) severe damage at 600 W, and (**e**) an anomalous case of minor damage at 600 W, (**f**) representative modes for each cluster, (**g**) sonography captured immediately after the 60th HIFU pulse, (**h**) histological image with damage grading (green arrows: vessels), (**i**) a cumulative Doppler power map superimposed onto the sonography, (**j**) cumulative Doppler power superimposed onto the histological image, and (**k**) contour map stretched axially by 20% to compensate for the pressure applied during the treatment. Courtesy of [59].

**Table 1:** Summary of sonography applications in the entire HIFU procedure.

Task		Advantages	Shortcomings
tumor diagnosis		enhanced tumor visualization, superior sensitivity and specificity	complexity of MU and multimodal imaging modalities in practice
treatment planning		quantitative evaluation of acoustic window, automatic parameter setting	3D quantification still infeasible, accuracy of planning needs improvement
localization & navigation	image fusion	improved image guidance significantly	limited adoption in clinics
	interference removal	technically feasible and effective	no clinical evaluation
monitoring	lesion detection	promising results with enhanced accuracy, sensitivity, and specificity	best approach unknown, no clinical and complete evaluation
	thermometry	solution becoming available	no comparison with MRI thermometry, limited maximum temperature range and accuracy, calibration required
	cavitation detection	providing quantitative, 2D metrics	limited spatiotemporal resolution
outcome evaluation		accurate lesion assessment	more physiological and pathological information required
follow-up & prediction		already in wide practice, prediction available	little prediction evaluation and simple model structure

## Data Availability

No new data were created or analyzed in this study. Data sharing is not applicable to this article.

## Abbreviations

The following abbreviations are used in this manuscript:

HIFU	High-intensity focused ultrasound
MRI	Magnetic resonance imaging
US	Ultrasound
MRgHIFU	MRI-guided HIFU
USgHIFU	US-guided HIFU
CEUS	Contrast-enhanced ultrasound
MB	Microbubble
NB	Nanobubble
AVD	Acoustic vaporization droplet
MU	Multimodal ultrasound
ABVS	Automated breast volume scanning
SWE	Shear wave elastography
CT	Computed tomography
PET	Positron emission tomography
DII	Dysmenorrhea improvement index
AVSR	Adenomyosis volume shrinkage ratio
ML	Machine learning
CNN	Convolutional neural network
DSC	Dice similarity coefficient
SVD	Singular value decomposition
PI	Pulse inversion
HMIgFUS	Harmonic motion imaging guided focused ultrasound
CNR	Contrast-to-noise ratio
FRPCA	Frequency-domain robust principal component analysis
SSIM	Structural similarity
DL	Deep learning
MPS	Minimum-peak-sidelobe
PIS	Pulse inversion subtraction
RF	Radio frequency
IBS	Integrated backscatter
ROC	Receiver-operating characteristic
IMF	Intrinsic mode functions
EMD	Empirical mode decomposition
CS	Compressive sensing
TA	Twinkling artifact
ASE	Axial strain elastogram
ASSE	Axial-shear strain elastogram
HMI	Harmonic motion imaging
FUS	Focused ultrasound
GPU	Graphics processing unit
DAS	Delay-and-sum
CPU	Central processing unit
CSR	Contrast-to-speckle ratio
WUE	Weighted ultrasound entropy
BSA	Bovine serum albumin
hNSE	Horizontally normalized Shannon entropy
DACI	Differential attenuation coefficient intercept
DSI	Dense speed-of-sound shift imaging
CBE	Change in backscattered energy
ROI	Region of interest
BMTS	Breath-guided multimodal teacher-student
MMAM-TSD	Multimodal attention multiscale teacher-student knowledge distillation
PCD	Passive cavitation detection
SVM	Support vector machine
SNR	Signal-to-noise ratio
DMD	Dynamic mode decomposition
PAM	Passive acoustic mapping
PCF	Phase coherence factor
CMF	Cross-spectral matrix fitting
FWHM	Full width at half maximum
RCB	Robust capon beamformer
spTV	Sparse total variation
ElNet	Elastic net
mpMRI	Multiparametric MRI
TRUS	Transrectal ultrasound
DCA	Decision curve analysis
APDT	Arterial-phase perfusion delay time
OMCLF	Optimized multi-task contrastive learning framework
SSL	Self-supervised learning
GA	Genetic algorithm
MWPA	Multi-wavelength photoacoustic
AO	Acousto-optic
